# Focal and Segmental Glomerulosclerosis and Membranous Nephropathy overlapping in a patient with Nephrotic Syndrome: a case report*

**DOI:** 10.1590/2175-8239-JBN-2018-0239

**Published:** 2019-02-25

**Authors:** Crislaine Aparecida da Silva, Fabiano Bichuette Custódio, Maria Luíza Gonçalves dos Reis Monteiro, Stanley de Almeida Araújo, Liliane Silvano Araújo, Rosana Rosa Miranda Côrrea, Marlene Antônia dos Reis, Juliana Reis Machado

**Affiliations:** 1 Universidade Federal do Triângulo Mineiro, Instituto de Ciências Biológicas e Naturais, Uberaba, MG, Brasil.; 2 Universidade Federal de Minas Gerais, Hospital das Clínicas, Belo Horizonte, MG, Brasil.

**Keywords:** Glomerulosclerosis, Focal Segmental, Glomerulonephritis, Membranous, Nephrotic Syndrome, Glomerulosclerose Segmentar e Focal, Glomerulonefrite Membranosa, Síndrome Nefrótica

## Abstract

**Introduction:**

Some cases of membranous nephropathy (MGN) present focal segmental glomerulosclerosis (FSGS) typically associated with disease progression. However, we report a case of a patient who seemed to have MGN and FSGS, both primary.

**Case presentation:**

A 17-year-old female, Caucasian, presenting lower extremity edema associated with episodes of foamy urine and high blood pressure, had physical and laboratorial exams indicating nephrotic syndrome. A renal biopsy was performed and focal and segmental glomerulosclerosis were observed under light microscopy in some glomeruli presented as tip lesion, and in others it was accompanied by podocyte hypertrophy and podocyte detachment in urinary space, compatible with podocytopathy FSGS. Besides, there were thickened capillary loops with basement membrane irregularities due to "spikes" compatible with MGN stage II. Immunofluorescence showed finely granular IgG, IgG4, and PLA2R deposits in capillary loops and, in electron microscopy, subepithelial deposits and foot process effacement. These morphological findings are compatible with FSGS and MGN stage II.

**Conclusions:**

In the present case, clinical and morphological characteristics showed a possible overlap of primary FSGS and MGN as focal and segmental glomerulosclerosis does not seem to be related with MGN progression but with the podocytopathy FSGS.

## Resumo

**Introdução:**

Alguns casos de nefropatia membranosa (NM) apresentam glomeruloesclerose segmentar e focal (GESF) tipicamente associada a progressão da doença. Contudo, relatamos o caso de uma paciente que parece ter NM e GESF, ambas primárias.

**Apresentação do caso:**

Uma jovem branca de 17 anos de idade com edema de membros inferiores associado a episódios de urina espumosa e hipertensão apresentou-se com achados físicos e laboratoriais sugestivos de síndrome nefrótica. Foi realizada biópsia renal. GESF foi observada por microscopia de luz em alguns glomérulos que apresentavam lesões de ponta, enquanto em outros o achado era acompanhado por hipertrofia podocitária e descolamento de podócitos no espaço urinário, compatíveis com podocitopatia GESF. Além disso, as alças capilares estavam espessadas com irregularidades na membrana basal devido a "espículas" compatíveis com NM estágio II. Imunofluorescência revelou depósitos finamente granulares de IgG, IgG4 e PLA2R nas alças capilares. Microscopia eletrônica exibiu depósitos subepiteliais e apagamento de pedicelos. Tais achados morfológicos são compatíveis com GESF e NM estágio II.

**Conclusões:**

No presente caso, as características clínicas e morfológicas revelaram uma possível sobreposição de GESF primária e NM, uma vez que a glomeruloesclerose segmentar e focal não parece estar relacionada com a progressão da NM, mas com a podocitopatia GESF.

## Introduction

Membranous nephropathy (MGN) is caused by the deposition of subepithelial immunocomplexes, accounts for about 20% of primary glomerulopathies in adults and predominantly affects men between 40-50 years old^1^. Patients present proteinuria, edema, hypoalbuminemia and hyperlipidemia, and the primary form (75%) is due to *in situ* immunoglobulin deposits against phospholipase A2 receptor (PLA2R) a podocyte antigen. In stage I of the disease, glomeruli are normal in light microscopy (LM) and diagnosis is possible only through immunofluorescence which showed IgG subepeithelial deposits (IF) and transmission electron microscopy (TEM), which evidence IgG subepithelial deposits. Stage II presents glomerular basement membrane (GBM) projections between deposits, named "spikes", better visualized by silver stain. As the disease progresses, deposits are incorporated in GBM, which results in a pinhole aspect (stage III) and are posteriorly reabsorbed, creating a thicker GBM (stage IV)^2^.

The coexistence of MGN with other renal diseases was already reported^3^^-^ 6 including MGN and FSGS 7 ^,^ 8, with FSGS cellular^9^ and collapsing variants 10 ^,^ 11. Therefore, we report the case of a patient with morphological characteristics of two primary glomerular diseases, MGN and FSGS.

## Case presentation

A 17 year-old female, Caucasian, investigating proteinuria, presented lower extremity edema associated with episodes of foamy urine and high blood pressure. The patient reported no use of medications, no arthralgia, arthritis, or skin lesions, and no family history of nephropathy. Laboratorial exams at admission showed serum creatinine: 0.8 mg/dL; urea: 22 mg/dL; glycemia: 82 mg/dL; total cholesterol: 460 mg/dL; LDL cholesterol: 340 mg/dL; triglycerides: 243 mg/dL; and serum albumin: 2.59 g/dL. Her proteinuria was 4370 mg/24h and urinalysis had 300 mg/dL of proteins, 10,000 leucocytes and 22,000 erythrocytes. Rheumatoid factor was 13 UL/mL and plasma complement particles C3 and C4 were normal. Dosages of FAN, anti-DNA, and serology for HIV, HCV, HBV, and VDRL were negative. After exams, she started using 40 mg/day furosemide and 20 mg/day Simvastatin.

**Figure 1:**
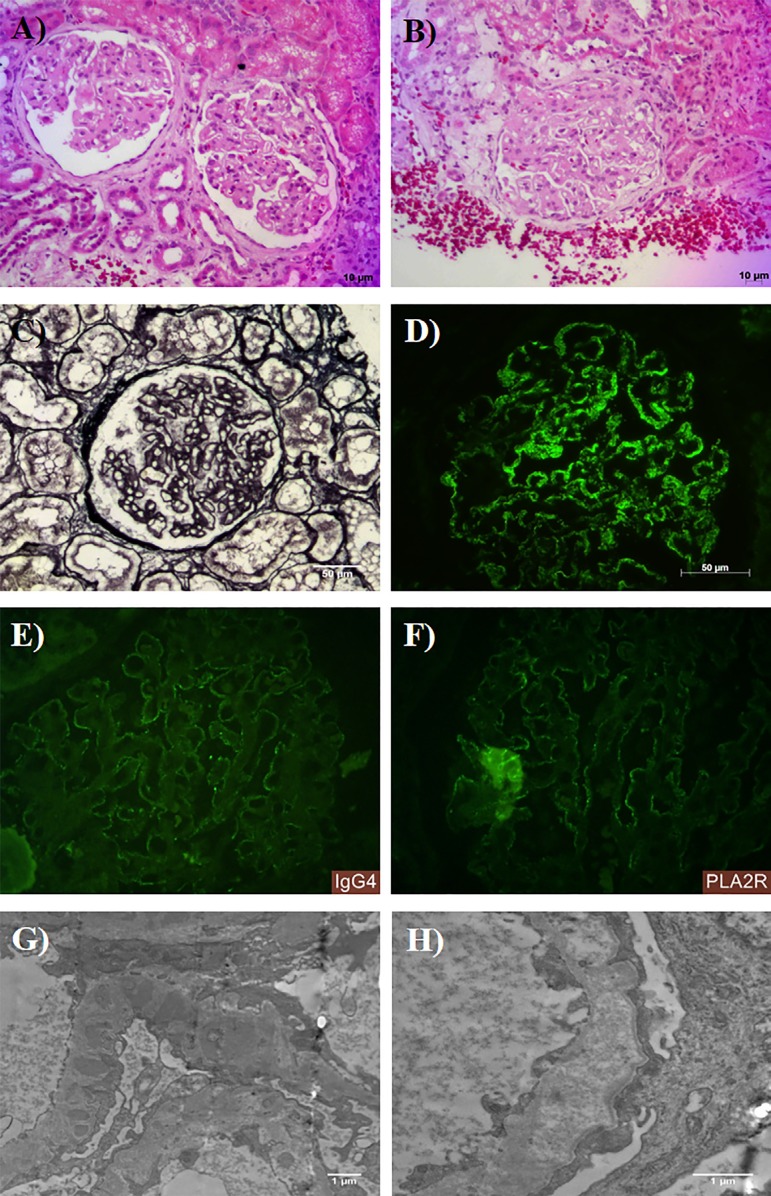
Histological sections of renal biopsy with morphological diagnosis of membranous glomerulopathy (MGN) concurrent with focal segmental glomerulosclerosis (FSGS). Light microscopy shows (A) thickening of capillary loops due to epimembranous deposits (HE). (B) Segmental sclerosis tip lesion, podocyte hypertrophy, and podocyte detachment in urinary space (HE). (C) Irregularities in glomerular basement membrane forming "spikes" (PAMS). Immunofluorescence for (D) IgG, (E) IgG4, and (F) PLA2R in a marked positive, finely granular, subepithelial, global and diffuse pattern. Electron microscopy showing a capillary loop with electron-dense subepithelial deposits with "spikes" formation in GBM (G) and foot process effacement with areas with deposits reabsorption (F).

The patient underwent renal biopsy without any intercurrence, and LM, IF, and TEM were performed. There were 23 glomeruli for analysis by LM. All glomeruli had thickened capillary loops due to epimembranous deposits (Figure 1A). There were also basement membrane irregularities forming "spikes" in a global and diffuse pattern in glomeruli (Figure 1B). Besides, 10 glomeruli had increased mesangial matrix with collapse of capillary loops in less than half of each glomerulus, characterizing segmental sclerosis; some had podocyte hypertrophy and podocyte detachment in urinary space, with a glomerulus showing tip lesion (Figure 1C). Interstitial fibrosis and tubular atrophy were mild. Vascular compartment was unremarkable.

IF showed marked granular global and diffuse staining for IgG, IgG4, and PLA2R, in glomeruli (Fig.1D), as well as Kappa and Lambda light chains. C3 had mild granular segmental positivity in glomeruli and segmental positivity in Bowman's capsule. IgA, IgM, C1q and fibrinogen were negative.

TEM showed electron-dense amorphous subepithelial deposits, with some deposits separated from each other by basement membrane spikes (Figure 1E), areas of immune complex reabsorption and foot process effacement (Fig.1F). There was no mesangial, subendothelial, or other glomerular deposits.

After the diagnosis was confirmed, treatment with angiotensin-converting enzyme inhibitor (ACEI) was started and the patient presented spontaneous remission for 1 year. After this period, there was an increase in proteinuria (5712 mg/24h) and cyclosporine treatment was started. Due to lack of response for 3 months, treatment with cyclophosphamide alternated with prednisone was started, with improvement of proteinuria.

## Discussion

This is a case report of a patient with nephrotic syndrome (NS), which renal biopsy showed morphological pattern of two different diseases. GBM irregularities by LM, IgG, and PLA2R deposits by IF, and subepithelial electron-dense deposits by TEM are characteristic of membranous nephropathy stage II 2. In addition, LM findings of focal and segmental glomerulosclerosis, podocyte hypertrophy and tip lesion, and TEM findings of foot process effacement characterize the podocytopathy FSGS 12. There was no evidence of secondary diseases.

MGN and FSGS are important causes of NS in adults, MGN being responsible for 20% of cases and FSGS for 40% of cases. MGN pathogenesis involves the formation of immunocomplexes in subepithelial region and it is considered an autoimmune disease limited to the kidney as the immunocomplex formation is due to immunoglobulin binding to podocytary antigen PLA2R *in situ*, which leads to activation of complement system, causing podocytary injury and consequently NS and renal failure^13^. FSGS is a podocytopathy in which glomerular injury is caused by intrinsic and/or extrinsic changes in podocytes, which leads to foot process effacement and sclerosis^12^.

MGN has a widely variable clinical course, as there may be patients with spontaneous remission of proteinuria, persistent proteinuria, and progression to renal failure^1^. Glomerulosclerosis is a common finding of MGN and these patients usually have a higher blood pressure, longer duration of proteinuria, persistent hematuria, and higher creatinine levels compared to patients without this finding. Besides, marked interstitial fibrosis and tubular atrophy and the presence of atherosclerosis are observed, indicating a poorer prognosis for these patients, which probably represents an evolution of MGN to chronicity. Most of these cases have MGN in more advanced stages, such as III and IV, since glomerulosclerosis could be due to injuries in podocytes as a result of disease progression itself, generating active and degenerative changes in these cells, leading to adhesion to parietal epithelium and development of sclerosis^14^^,^ 15.

Although glomerulosclerosis in MGN may be related to the natural progression of the disease, sclerosis in this case is probably not related to epithelial damage caused by progression of disease to advanced stages, but most likely represents a morphologic characteristic of the primary glomerular disease FSGS. Therefore, this patients most probably have primary FSGS and primary MGN as two overlapping diseases, as segmental sclerosis focally as tip lesion, glomeruli with podocyte hypertrophy, and detached podocytes in urinary space, strongly suggest segmental sclerosis due to podocytopathies^16^. In contrast, segmental sclerosis due to scars generally present as sclerosis with hypocellular areas and adhesion to Bowman's capsule.

This fact corroborates the literature that shows about 78% of the patients with FSGS with MGN present segmental sclerosis with hypertrophic podocytes in addition to hypertension, hematuria, and higher proteinuria when compared to patients with MGN without sclerosis^17^.

A case of a young female with a similar age to our patient, in whom segmental sclerosis accompanied by podocyte hypertrophy, finely granular IgG deposition and foot process pedicels effacement was observed, suggested the concomitance of the two entities^9^.

Our patient presented a primary MGN-compatible antibody marking profile, since PLA2R and IgG4 deposition were found in the biopsy. It has recently been observed that patients with MGN combined with FSGS present clinical and autoantibodies profiles compatible with primary MGN. Approximately 80% of MGN-FSGS patients presented circulating PLA2R, similar to those with MGN alone. In patients with FSGS alone, PLA2R screening was negative. Likewise, 75% of the patients with combined lesions and 79% of the MGN patients had positive PLA2R glomerular expression. In addition, patients presenting MGN with or without sclerosis had glomerular deposition of IgG4 in contrast to patients with only FSGS^18^.

Regarding clinical evolution, after one year, our patient entered spontaneous remission. Then, the disease progressed to baseline proteinuria of 2.655 mg/24h, until the present moment. It has been reported that the presence of sclerosis in MGN is related to worse prognosis^19^. After a 3-year follow-up, 64 patients with combined lesions presented worse evolution than patients with only MGN, and this was not related to more advanced stages of MGN^20^. This shows that the occurrence of the two overlapping diseases may predict the clinical course of patients with MGN regardless of staging.

In view of our findings and literature descriptions, we conclude that FSGS and MGN can be superimposed, but the mechanism of concomitance is not known. An initial glomerular injury can predispose the onset of an immunocomplex-mediated disease due to injury to the glomerular filtration barrier^13^. Thus, damage to podocytes in FSGS can lead to subepithelial immunocomplex formation by exposure of local antigens, as observed in a patient in whom, after 7 years of diagnosis of FSGS, had MGN in a second biopsy^8^, indicating primary lesion of FSGS may have led to the development of MGN. In addition, MGN's injuries may lead to the emergence of FSGS, as subepithelial deposits may hinder the adhesion of podocytes to GBM through α3β1 integrin, leading to podocyte loss. Denuded areas of GBM favor the adhesion of these regions to parietal epithelium with synechiae formation, capillary obliteration, and later sclerosis, developing segmental glomerulosclerosis^15^.

Some cases of MGN present focal segmental glomerulosclerosis typically associated with disease progression. However, we report a case of a patient who seemed to have FSGS and MGN, both primary, as morphological characteristics of biopsy and clinical evolution strongly suggested the concomitance of the two primary glomerular diseases. Nevertheless, the mechanisms of injury in these cases are still uncertain. Therefore, more studies are needed to elucidate the overlap of these primary glomerulopathies.
